# Avian Viruses that Impact Table Egg Production

**DOI:** 10.3390/ani10101747

**Published:** 2020-09-25

**Authors:** Mohamed S. H. Hassan, Mohamed Faizal Abdul-Careem

**Affiliations:** 1 Faculty of Veterinary Medicine, University of Calgary, Health Research Innovation Center 2C53, 3330 Hospital Drive NW, Calgary, AB T2N 4N1, Canada; msh.hassan@ucalgary.ca; 2 Department of Poultry Diseases, Faculty of Veterinary Medicine, Assiut University, Assiut 71515, Egypt

**Keywords:** layer, viral infection, reproductive tract, egg production, eggshell quality, internal egg quality

## Abstract

**Simple Summary:**

Global table egg production is a multibillion-dollar industry. Poultry eggs provide an important source of protein, and consumers demand high-quality products. The industry relies on efficiency and good management, including vaccinations against the major poultry diseases, in order to ensure that they can match consumer demand. However, although there are a number of effective disease prevention protocols in place on poultry farms, viruses that affect egg production continue to be a major constraint for the sustainability of the table egg industry globally. Various factors related to either the infecting virus, the host, or the management system have been demonstrated to contribute significantly to the challenge for effective egg production. Viral infections in laying flocks have been shown to target various biological mechanisms that are essential for the quantitative and qualitative traits of egg production. This review describes the mechanisms involved in viral diseases of poultry that induce undesirable effects on egg production.

**Abstract:**

Eggs are a common source of protein and other nutrient components for people worldwide. Commercial egg-laying birds encounter several challenges during the long production cycle. An efficient egg production process requires a healthy bird with a competent reproductive system. Several viral pathogens that can impact the bird’s health or induce reversible or irreversible lesions in the female reproductive organs adversely interfere with the egg industry. The negative effects exerted by viral diseases create a temporary or permanent decrease in egg production, in addition to the production of low-quality eggs. Several factors including, but not limited to, the age of the bird, and the infecting viral strain and part of reproductive system involved contribute to the form of reproductive disease encountered. Advanced methodologies have successfully elucidated some of the virus–host interactions relevant to the hen’s reproductive performance, however, this branch needs further research. This review discusses the major avian viral infections that have been reported to adversely affect egg productivity and quality and aims to summarize the current understanding of the mechanisms that underlie the observed negative effects.

## 1. Introduction

Eggs are an inexpensive and important source of nutritive elements, including protein, essential vitamins, and minerals, and, as such, are included in the daily diet of many [1]. The global production of eggs has been expanding over the years, reaching approximately 82.8 million tons of eggs in 2018, and further increase is expected [2]. Modern extensive poultry production systems have developed to provide the bird’s needs of appropriate nutrition, ventilation, and artificial lighting, and have adopted sound biosecurity measures for the purpose of achieving maximum productivity. Nevertheless, a large burden of viral diseases could compromise the reproductive efficiency of the confined layers, leading to adverse effects on egg output and quality (Table 1). In the mature domestic hen, an estrogen-based mechanism was proposed to explain the development of a single, left side reproductive tract (ovary plus oviduct) [3]. Each part of the hen’s reproductive tract exerts a role in egg formation. The first component of the oviduct is known as the infundibulum and it receives the ovum, and this is the site of fertilization. Within fifteen minutes of receiving the ovum, the yolk with the ovum moves to the magnum, where the egg albumen is deposited. Usually, the albumen deposition process takes about three hours. Following the albumen deposition, the egg moves to the isthmus, where the inner and outer shell membranes are formed, in addition to adding water and minerals. The latter process takes about an hour, and shell formation takes place in the uterus or shell gland, where the egg persists for up to 21 h. Finally, the fully formed egg passes through the vagina to the cloaca to be released outside of the hen’s body.

Unfavorable effects on egg production are encountered by viral pathogens that either induce pathologies in the reproductive organs (Table 2) or indirectly affect the bird’s health. Despite the importance of egg production, reviews that focus on viral diseases that impact egg production and quality are scarce. The current review focuses on a description of the various reproductive forms of the major viral diseases of poultry. Emphasis will be on the underlying mechanisms of how viral pathogens induce undesirable effects on egg production. Accordingly, the major viral infections that influence egg production and quality can be classified into two broad categories—viral infections with a direct influence on the reproductive tract and viral infections with an indirect impact on egg production.

## 2. Viral Infections that Induce Ovary and/or Oviduct Disease

### 2.1. Infectious Bronchitis (IB)

Infectious bronchitis virus (IBV), the causative agent of IB, is a gammacoronavirus in the family *Coronaviridae*, which exists as multiple heterologous strains [31]. Although IBV initially infects the respiratory system of chickens, some strains have a genital tropism. The extent of the reproductive disease may differ with the bird’s age and the strain of the causative virus [32].

In layers, infection at peak production leads to a severe fall in egg production, accompanied by a reduction in shell quality and a watery albumen. During the necropsy examination, a reduced length and weight of the oviduct, as well as regression of the ovaries were observed [18]. Experimental infection with an Arkansans (Ark) strain of IBV contributed to a considerable loss of internal and external egg quality [33]. Recently, Massachusetts (Mass) type IBV was shown to be responsible for repeated outbreaks of shell-less egg syndrome (SES) in Western Canada [34]. An increase in the anti-IBV antibody titer was detected in non-vaccinated laying flock with a history of eggshell apex abnormalities (EAA) [35]. A threshold of 6 log2 for the hemagglutination inhibition (HI) anti-IBV antibody titer was shown to be protective against the negative effects on egg production [36]. The production of pale or inconsistently colored eggs could be contributed by IBV-induced lesions in the uterus, which decrease the deposition of the main eggshell pigment, protoporphyrin IX [37]. IBV injection into the magnum lumen was accompanied by a down-regulation in the expression of the genes related to the formation of eggshell (collagen type I and calbindin-D28 K) [38]. The glandular hypoplasia of the magnum, induced by IBV infection, could result in a reduction in the synthesis of albumen proteins and, consequently, a watery albumen and reduced egg weight [39]. Similarly, two Australian IBV strains that displayed an upper reproductive tract tropism [40] had a negative impact on the internal quality of the egg [41]. IBV infection has been revealed to be dependent on the binding of the spike (S) glycoprotein to the alpha 2,3-linked sialic acids on the avian cells [42,43]. However, the primary attachment of S1 proteins to the sialic acids on the surface of the oviduct epithelium did not explain the varying pathogenicity of IBV strains QX and B1648 to the oviduct [44].

Infection of female chickens within the first few days of life can cause detrimental lesions in the developing reproductive tract. Cystic dilatation in the oviduct, which has been detected in false layers with a reduced peak in egg production, could be a consequence of IBV exposure at one day old [19,20,21]. Despite the defects in the oviduct caused by early infection with IBV, these non-laying birds have commonly had fully functioning ovaries [19,45]. Infection of three-week-old chickens induced histopathological lesions in the ovaries three days post infection (dpi); however, no gross lesions were detected in the ovaries of mature birds [46]. Compared with healthy flocks, IBV infection in young chicken flocks resulted in a shorter peak egg-laying period and a lower mean egg weight [47]. Age-dependent resistance to IBV-induced reproductive tract abnormalities was reported [48,49].

### 2.2. Egg Drop Syndrome (EDS)

Egg drop syndrome virus (EDSV) is a member the genus *Atadenovirus* of the *Adenoviridae* family. Ducks and geese are believed to be the natural hosts, but disease outbreaks commonly impact laying chickens [24]. A fall in production and abnormal eggs are commonly described in naturally occurring outbreaks. Both experimental and natural infections are generally not accompanied by clinical signs, except for slight diarrhea [7,50,51,52]. Egg discoloration that commonly preceded the production of thin-shelled, soft-shelled, and shell-less eggs characterized the early outbreaks reported in the 1970s [7,51,53]. Naturally occurring outbreaks usually lasted 4 to 10 weeks, with a fall in egg production of up to 50% [6,7,51]. Adverse effects on the internal egg quality were not consistent between reports [7,50,51,52,54]. Research investigating the pathogenesis of EDSV infection established the pouch shell gland as the primary site of viral replication, which was associated with the production of abnormal eggs [55]. The lesions were minimal in mature chickens; however, uterine edema was observed [22,23]. It was believed that pathological changes in the uterus of the infected bird may have interfered with the proper formation of the eggshells [50].

### 2.3. Avian Metapneumovirus (AMPV) Infection

Avian Metapneumovirus was classified recently as the type species of the genus *Metapneumovirus* of the family *Pneumoviridae* [56]. The term turkey rhinotracheitis (TRT) has been used to describe clinical respiratory disease in turkeys. In particular, manifestations suggest upper respiratory tract infection, include snicking, rales, sneezing, nasal discharge, foamy conjunctivitis, and swollen infraorbital sinus [57]. Milder clinical signs are usually appreciated in chickens; however, swollen head syndrome (SHS) is thought to develop as a result of secondary bacterial infections [58].

Infection in laying birds usually contributes to a decrease in egg production and to reduced egg quality. Laying hens experimentally infected by the intravenous route, but not the oculonasal route, experienced a significant drop in the production of eggs, and an increased number of soft or thin-shelled eggs [8,59]. Egg peritonitis was described in the experimental infections of chickens and turkeys [8,60]. Lesions identified in the oviduct and ovary, including inspissated yolk or albumen in the oviduct, ovarian hemorrhages, and regression of ovary and oviduct, in addition to the detection of AMPV antigen in the oviduct, suggest a direct effect of the infection on the reproductive performance [8,60,61]. The pathogenicity of AMPV for the chicken’s oviduct was shown to be higher in the uterus than in the magnum and isthmus, which suggests a different susceptibility of epithelial cells for the three parts of the chicken’s oviduct [62]. In China, a similar disease picture was reported in egg-laying Muscovy ducks [63]. In Italy, longitudinal studies and sporadic samplings indicated that most pullet flocks get infected with AMPV before the laying begins [64].

### 2.4. Newcastle Disease (ND)

Avian orthoavulavirus 1 belongs to the family *Paramyxoviridae*, whereas the virus has been previously known as Newcastle disease virus (NDV) [56,65]. It infects a wide variety of avian species and represents one of the most important poultry pathogens [66]. The clinical signs vary in susceptible birds depending on the host species, virulence of the infecting strain, and immune status of the host. The experimental infection of chickens has identified four pathotypes, namely, velogenic, mesogenic, lentogenic, and asymptomatic enteric [67]. Respiratory and nervous manifestations with an elevated mortality are commonly observed after the infection of non-vaccinated birds with virulent NDVs [68,69].

In countries where ND is endemic, heavy vaccination programs are applied to face challenge of the disease [70]. Nevertheless, virulent strains of NDV remain isolated sporadically from vaccinated flocks [71,72]. A decreased egg production was reported in vaccinated layers challenged with a virulent NDV [73]. A study that investigated the epidemiology of ND in 31 commercial chicken farms in Eastern China recorded a 15–40% drop in the rate of egg production [74]. The production of abnormal eggs, including soft-shell eggs and spotted-shell eggs, was also described [10]. Challenged specific-pathogen-free (SPF) hens demonstrated small and flaccid oviducts and inactive ovaries during necropsy examination. The uterus was reported to be the main target part of the oviduct for both live vaccine and virulent strains, which may explain the changes observed in the shell quality [25]. The drop-in egg production was thought to be related to a decreased serum phosphorus level as a consequence of ND-induced kidney damage [75]. Furthermore, the lower calbindin-D28k (CaBP-D28k), a calcium-binding protein, mRNA expression in the uteri of NDV-infected hens was suggested to have a role in the change in shell quality [76]. The HI anti-NDV antibody titer levels showed a high correlation with protection against the negative effects on egg production [10].

### 2.5. Avian Influenza (AI)

Avian influenza viruses (AIVs) are classified as members of the genus *Influenzavirus* A of the family *Orthomyxoviridae* [77]. Serological reactions to the surface glycoproteins, haemagglutinin (HA) and neuraminidase (NA), have been used for the subtyping of AIVs and, accordingly, sixteen subtypes of HA (H1–16) and nine subtypes of NA (N1–9) have been recognized [78]. Influenza viruses are notorious for antigenic variation and hundreds of different subtypes exist because of antigenic drift and shift [79]. A large variety of avian species, both domestic and wild birds, have been shown to be susceptible to natural infections with AIVs [80]. Poultry production has incurred huge impacts as a result of frequent outbreaks of either highly pathogenic avian influenza (HPAI) or low pathogenic avian influenza (LPAI) [81]. The clinical signs that accompany infections with AIVs vary widely depending on the host species, age, immune status of the host, and the virus subtype involved. In chickens, for example, LPAI viruses developed disorders with mild to severe manifestations in respiratory, digestive, urinary, and reproductive systems. A sudden increase in mortality, up to 90%, may be the only indication of infection with HPAI viruses [82].

Losses from reduced egg production have been encountered as a result of AIV infections. An outbreak of AI that involved three closely-located laying farms resulted in a significant decrease (from 80 to 13%) in egg production and a total mortality rate of 69% [83]. Nonpathogenic H7N2 AIV was isolated from 10 commercial leghorn laying flocks in Pennsylvania, USA, which had a mild form of the disease with a total mortality of >4% and an egg production fall of 2% to 4%. Egg yolk peritonitis and salpingitis with edema in the oviduct were observed. However, the possibility that the lesions observed in the oviduct could be due to secondary bacterial infection could not be excluded [26]. In a study that investigated the significance of the widely distributed H9N2 as a primary pathogen in laying birds, infection caused a long-term decline in egg production. In addition, the study demonstrated that the virus replication-induced lesions were mainly seen in the infundibulum, which limits the reproductive functionality of the bird [27]. A decline in the production of eggs and a decreased shell thickness in the hens infected with LPAI H9N2 was shown to be coincidental with a decrease in CaBP-D28k mRNA expression in the uterus [84]. Experimental infection with a Belgian H3N1 LPAI strain induced very mild clinical signs in four-week-old SPF males compared with a 58% mortality and a complete cessation in egg production encountered in 34-week-old SPF layers. It was concluded that virus replication in the oviduct was a major component in disease development [11]. Comparing the daily recorded mortality and egg production rates to the pre-assigned thresholds showed a promising result for the early reporting of HPAI and LPAI outbreaks in commercial layer flocks in Netherland [85].

### 2.6. Marek’s Disease (MD)

Marek’s disease is a lymphoproliferative disease of chickens caused by strictly cell-associated alphaherpesvirus, which was recently assigned to the genus *Mardivirus* within the family *Herpesviridae* [86,87]. Live-vaccine-based control of MD is heavily practiced in laying flocks [88]. In a field study that lasted 18 months, vaccination reduced the number of birds from which the MD virus could be isolated, and the number of eggs produced per hen per day was found to be 4% higher in the vaccinated birds compared with the non-vaccinated birds [89]. Over a 10-year scope, an analysis of the effect of MD on egg production showed that the infected laying hens incurred a 5% egg production loss in comparison with the uninfected laying-hens [16]. The prolonged course of the disease, associated unthriftness, and lymphodegenerative syndromes that may involve the gonads (especially the ovary) could all act as factors in the recorded production losses [28,90].

### 2.7. Avian Encephalomyelitis (AE)

The egg transmitted avian encephalomyelitis virus (AEV) is a member of the *Picornaviridae* family [91]. In young chickens, the neurological nature of the disease involves clinical signs, such as ataxia, paralysis, and tremors [92]. However, clinical AE has been encountered in layer pullets following the application of live AE vaccines [93,94]. Naturally occurring outbreaks have also been identified in pheasants, quails, pigeons, and turkeys [95].

In laying birds, AE can negatively influence egg production and hatchability. Avian encephalomyelitis was diagnosed in breeding hens that experienced a transient decline in egg production. Additionally, a significant late embryonic mortality in the fertile eggs produced by these hens was reported [96]. In a commercial laying flock, a dramatic fall in egg production (up to 75%), lasting for 2 weeks, was associated with encephalomyelitis [13]. However, other naturally occurring outbreaks in non-vaccinated laying flocks showed lower rates (7.5% to 18%) of fall in egg production [97,98]. The infected adult birds did not exhibit neurological disorders that usually appeared in the chicks hatching from eggs produced around and during the time of the egg drop [96,99]. Virus replication in the ovary could be the most probable cause of the temporary decrease in egg production [100,101].

### 2.8. Other Viral Infections

Avian hepatitis E virus (HEV) belongs to *Orthohepevirus* B species within the *Hepeviridae* family [102]. In layer and broiler-breeder chickens, the virus has been associated with big liver and spleen (BLS) disease and hepatitis-splenomegaly (HS) syndrome [103,104]. The two diseases have often been correlated with increased mortality (1–4%) and reduced egg production (10–45%) in broiler breeder and laying hens [14,105,106,107]. Apart from the characteristic hepatosplenomegaly, oviduct regression was also observed [30]. In Hungary, the hatchability of the broiler breeder affected flocks was 36% to 46% lower than the expected level [108].

Duck hepatitis A virus (DHAV) is known to cause a highly contagious and acute disease in young ducklings, which is often associated with the sudden onset of a high mortality [109]. DHAV was assigned to the genus *Avihepatovirus* in the family *Picornaviridae* [110]. DHAV was recently isolated from the laying flocks of ducks in Eastern China that had egg drops and ovary-oviduct disease. The egg drop disease was experimentally reproduced with the isolated DHAV strain. Ovarian hemorrhage and necrosis were observed in the infected ducks [111].

## 3. Viral Infections that Compromise the General Physiological Status of the Bird Leading to Egg Production Problems

### 3.1. Infectious Laryngotracheitis (ILT)

The causative agent, *Gallid herpesvirus type 1* (GaHV-1), belongs to the genus *Iltovirus*, subfamily *Alphaherpesvirinae* of the *Herpesviridae* family [112]. ILT is an upper respiratory disease of chickens, which is characterized by mild or severe respiratory manifestations accompanied by production losses [113]. Like other members of the *Herpesviridae* family, GaHV-1 can establish latency in clinically recovered birds [114]. Stressors such as rehousing, and an onset of laying could trigger the re-excretion of the virus in recovered chickens [115].

A rapidly spreading ILT outbreak in a multiage (from 40 to 107 weeks) laying farm resulted in a decrease in egg production (up to 58%) and a slight increase in mortality [15]. In a survey that investigated the occurrence of ILT in 25 different layer flocks, the egg production, but not the mean egg weight, was lower in the infected flocks (16 flocks) [116]. As the gross and microscopic lesions were confined to the respiratory tissues, it was reasonable to infer that the fall in egg production was a secondary effect to the impaired heath status of the infected bird [117].

### 3.2. Avian Leukosis

Avian leukosis virus (ALV) infection of chickens is a substantial cause of economic losses because of tumor-associated mortalities and negative effects on production [118]. ALV is the type species of the genus *Alpharetrovirus* within *Retroviridae* family [119]. ALV shedding into the eggs was associated with lower egg production, delayed onset of laying, small-sized eggs, thin-shell eggs, and reduced hatchability compared with non-shedding hens [120,121]. Moreover, strains of chickens demonstrated a major impact on the rate of virus shedding and, consequently, production traits [120]. Avian leukosis was diagnosed in commercial egg layer flocks (white leghorn breed) that demonstrated lower peaks of egg production (55% to 80%). During necropsy examination, a lack of ovarian activity and various visceral lymphomas were observed in non-laying birds [122]. In a study that investigated the effect of the type of infection (congenital or horizontal) on egg production, contact-infected hens produced 28 more eggs per hen than those congenitally infected [123]. Important production criteria, including egg production rate, egg weight, and shell thickness, were negatively affected in chickens that harbored endogenous viral (ev) genes of ALVs in their genomes [124]. ALV subgroup J, associated with extreme losses in meat-type chickens since the late 1980s, was first reported to cause myeloid leukosis in commercial layer flocks in 2002 [125]. Since then, several reports have described a dramatic reduction in egg production accompanied by ALV-J induced tumors in egg-type chickens [126,127].

### 3.3. Fowlpox

Fowlpox is a common poultry viral disease and is characterized by cutaneous nodular lesions and/or fibronecrotic lesions on the mucous membranes of the upper respiratory and/or digestive tracts [128]. Avianpox viruses are members of the *Avipoxvirus* genus in the family *Poxviridae* [129]. The decrease in the production of eggs in laying birds is as a result of the poor conditions of infected birds and the intensity of the proliferative lesions [130]. Egg production dropped to 15% in a small flock of brown layers that experienced an outbreak of both cutaneous and diphtheritic forms of fowlpox [131].

## 4. Conclusions

Over the past few decades, intensive poultry production has been shown to be a double-edged sword. Raising large numbers of birds in a confined housing system has been successful in addressing the ever-increasing needs for white meat and eggs. However, this situation created an environment for pathogenic microorganisms to evolve and cause serious threats for the industry. Viral pathogens with a tropism for the reproductive organs or those that cause debilitating health conditions demonstrated mild to severe adverse effects on the egg production process. Multiple factors, mainly including host species, strain of the virus, and age and immune status of the host, all play a major role in the type of reproductive disease encountered. Using advanced molecular, microscopy, and virus culture and detection approaches would help in further understanding the pathogenesis of reproductive diseases caused by these viral infections. Future directions would be to explore the mechanisms that underlie the varying tropisms of different strains of the same virus to the reproductive organs, varying susceptibility of different parts of the reproductive tract to the viral infection, and the influence of the genetic make-up of the birds. Analytical and biochemical approaches would help us to understand the systemic changes that could have adverse impacts on egg production and quality.

## Figures and Tables

**Table 1:** Adverse effects on egg production and quality observed in different viral diseases.

Viral Disease	Drop in Egg Production (%)	References	Egg Quality Issues	References
Infectious Bronchitis (IB)	Up to 70%	[4]	Misshapen eggs, rough and thin shells, eggshell discoloration, and watery albumen	[5]
Egg Drop Syndrome (EDS)	Up to 50%	[6]	Pale eggshell, thin, soft, and shell-less eggs and watery thin albumen	[7]
Avian Metapneumovirus (AMPV) Infection	Up to 60%	[8]	Soft or thin-shelled eggs	[8]
Newcastle Disease (ND)	Up to 100%	[9]	Decreased shell thickness, soft shells, spotted shells, and decreased albumen height	[9,10]
Avian Influenza (AI)	Up to 100%	[11]	Misshapen, discolored, and fragile eggs	[12]
Avian Encephalomyelitis (AE)	Up to 75%	[13]	-	-
Avian Hepatitis E Virus (HEV) Infection	Up to 45%	[14]	-	-
Infectious Laryngotracheitis (ILT)	Up to 58%	[15]	-	-
Marek’s Disease (MD)	Up to 5%	[16,17]	-	-

**Table 2:** Lesions in reproductive organs observed in different avian viral diseases.

Viral Disease	Lesions in Reproductive Organs	References
Infectious Bronchitis (IB)	Ovarian regression and atrophied oviduct	[18]
	Cystic lesions in the oviduct	[19,20,21]
Egg Drop Syndrome (EDS)	Uterine edema	[22,23]
	Inactive ovaries	[24]
Avian Metapneumovirus (AMPV) Infection	Ovarian hemorrhages, inspissated yolk in shell gland and regression of ovary and oviduct	[8]
Newcastle Disease (ND)	Small and flaccid oviducts and inactive ovaries	[25]
Avian Influenza (AI)	Salpingitis	[11,26,27]
Marek’s Disease (MD)	Ovarian tumors	[28,29]
Avian Hepatitis E Virus (HEV) Infection	Oviduct regression	[30]
